# Supplementary material for: Depression and incidence of inflammation-related physical health conditions: a cohort study in UK Biobank
Source: BMC Psychiatry. 2025 Oct 2;25:922. doi: 10.1186/s12888-025-07337-7 (PMC12490120; doi:10.1186/s12888-025-07337-7)
Supplement: Supplementary file 1 — Supplementary Material 1 [file 12888_2025_7337_MOESM1_ESM.pdf]

Additional File

Table S1: Availability periods of the electronic health records

Data source	Region	Data complete
Hospital admission	Scotland	Earliest record: Jan 1981<br>Complete to: 31 Aug 2022
	Wales	Earliest record: Jan 1998*<br>Complete to: 31 May 2022
	England	Earliest record: Apr 1997<br>Complete to: 31 Oct 2022
Cancer registry	Scotland	Earliest record: 1957<br>Complete to: 30 Nov 2021
	Wales	Earliest record: 1971<br>Complete to: 31 Dec 2016
	England	Earliest record: 1971<br>Complete to: 31 Dec 2020
Primary care	Scotland	Earliest record[1]: Aug 1937<br>Complete to: 31 Mar 2017
	Wales	Earliest record[#]: May 1940<br>Compete to: 31 Aug 2017
	England (TPP)	Earliest record[1]: Dec 1937<br>Complete to: 31 May 2016

* At the time of publication, the UK Biobank website (https://biobank.ndph.ox.ac.uk/showcase/exinfo.cgi?src=Data_providers_and_dates) lists the start date for Wales as 1991, but data records are not consistently available until January 1, 1998

# Records prior to each participant's date of birth were excluded.

Table S2: Long-term health conditions measured at baseline

Condition type	Condition	Condition includes
Physical	Benign neoplasm of brain and other CNS	
	Haematological malignancies	Hodgkin lymphoma
		Leukaemia
		Monoclonal gammopathy of undetermined significance (MGUS)
		Multiple myeloma and malignant plasma cell neoplasms
		Myelodysplastic syndromes
		Non-Hodgkin lymphoma
		Polycythaemia vera
		Haematological malignancy - other
	Non-melanoma skin malignancies	
	Solid organ malignancies	Primary malignancy - biliary tract
		Primary malignancy - bladder
		Primary malignancy - bone and articular cartilage
		Primary malignancy - brain, other central nervous system and intracranial
		Primary malignancy - breast
		Primary malignancy - cervical
		Primary malignancy - colorectal and anus
		Primary malignancy - kidney and ureter
		Primary malignancy - liver
		Primary malignancy - lung and trachea
		Primary malignancy - malignant melanoma
		Primary malignancy - mesothelioma
		Primary malignancy - multiple independent sites
		Primary malignancy - oesophageal
		Primary malignancy - oro-pharyngeal
		Primary malignancy - ovarian
		Primary malignancy - pancreatic
		Primary malignancy - prostate
		Primary malignancy - stomach
		Primary malignancy - testicular
		Primary malignancy - thyroid
		Primary malignancy - uterine
		Primary malignancy - other organs
		Secondary malignancy - adrenal gland
		Secondary malignancy - bone
		Secondary malignancy - bowel
		Secondary malignancy - brain, other central nervous system and intracranial
		Secondary malignancy - liver and intrahepatic bile duct
		Secondary malignancy - lung
		Secondary malignancy - lymph nodes
		Secondary malignancy - pleura
		Secondary malignancy - retroperitoneum and peritoneum
		Secondary malignancy - other organs
	Cardiomyopathy	Dilated cardiomyopathy
		Hypertrophic cardiomyopathy
		Other cardiomyopathy
	Conduction disorders and other arrhythmias	Atrioventricular block, complete
		Sick sinus syndrome
		Supraventricular tachycardia
	Coronary heart disease	Coronary heart disease not otherwise specified
		Myocardial infarction
		Stable angina
		Unstable angina
	Heart valve disorders	Multiple valve disorder
		Nonrheumatic aortic valve disorders

Condition type	Condition	Condition includes
		Nonrheumatic mitral valve disorders
		Rheumatic valve disorder
Physical	Stroke	Intracerebral haemorrhage
		Ischaemic stroke
		Stroke not otherwise specified
	Atrial fibrillation	
	Heart failure	
	Hypertension	
	Peripheral arterial disease	
	Primary pulmonary hypertension	
	Transient ischaemic attack	
	Chronic liver disease	Alcoholic liver disease
		Autoimmune liver disease
		Hepatic failure
		Liver fibrosis, sclerosis and cirrhosis
		Portal hypertension
		Chronic viral hepatitis
	Gastro-oesophageal reflux, gastritis and similar [abbreviated to gastro-oesophageal reflux disease (and similar) in the main text]	Barrett's oesophagus
		Gastritis and duodenitis
		Gastro-oesophageal reflux disease
		Oesophagitis and oesophageal ulcer
	Inflammatory bowel disease	Crohn's disease
		Ulcerative colitis
	Coeliac disease	
	Diverticular disease of intestine (acute and chronic)	
	Fatty liver	
	Irritable bowel syndrome	
	Peptic ulcer disease	
	Hearing loss	
	Meniere disease	
	Addison’s disease	
	Cystic fibrosis	
	Hypo or hyperthyroidism	
	Type 1 diabetes	
	Type 2 diabetes	
	Diabetes not otherwise specified	
	Glaucoma	
	Macular degeneration	
	Visual impairment and blindness	
	Chronic renal disease	Chronic kidney disease
		End stage renal disease
		Glomerulonephritis
		Tubulo-interstitial nephritis
	Erectile dysfunction	
	Hyperplasia of prostate	
	Non-acute cystitis	
	Urinary incontinence	
	Allergic and chronic rhinitis	
	Asbestosis	
	Asthma	
	Bronchiectasis	
	Chronic obstructive pulmonary disease	
	Sleep apnoea	
	Iron and vitamin deficiency anaemia	Folate deficiency anaemia
		Iron deficiency anaemia
		Vitamin B12 deficiency anaemia

Condition type	Condition	Condition includes
	Immunodeficiencies	
	Sarcoidosis	
	Sickle-cell anaemia	
	Thalassaemia	
	HIV	
	Tuberculosis	
Physical	Inflammatory arthritis and related conditions	Ankylosing spondylitis
		Juvenile arthritis
		Lupus erythematosus (local and systemic)
		Polymyalgia rheumatica
		Postinfective and reactive arthropathies
		Psoriatic arthropathy
		Rheumatoid arthritis
		Systemic sclerosis
	Osteoporosis and vertebral crush fractures	Collapsed vertebra
		Osteoporosis
	Gout	
	Osteoarthritis (excl spine)	
	Spinal stenosis	
	Peripheral or autonomic neuropathy	Diabetic neurological complications
		Disorders of autonomic nervous system
		Peripheral neuropathies (excluding cranial nerve and carpal tunnel syndromes)
	Cerebral palsy	
	Epilepsy	
	Migraine	
	Motor neurone disease	
	Multiple sclerosis	
	Myasthenia gravis	
	Parkinson's disease	
	Post-viral fatigue syndrome, neurasthenia and fibromyalgia	
	Down's syndrome	
	Psoriasis	
	Dementia	
Mental	Alcohol problems	
	Anorexia and bulimia nervosa	
	Anxiety disorders	
	Autism and Asperger's syndrome	
	Bipolar affective disorder and mania	
	Intellectual disability	
	Obsessive-compulsive disorder	
	Other psychoactive substance misuse	
	Post-traumatic stress disorder	
	Schizophrenia, schizotypal and delusional disorders	

Abbreviation: CNS, central nervous system

Table S3: Hazard ratios for the association of baseline depression, age, sex, sociodemographic, baseline comorbidities, and lifestyle factors with incident CHD (n=157,028)

**Variables**		**Hazard Ratio (95% CI)**			
		**Unadjusted**	**Model 1***	**Model 2****	**Model 3*****
**Depression**		1.15 (1.08, 1.22)	1.36 (1.28, 1.44)	1.33 (1.25, 1.41)	1.00 (0.94, 1.07)
**Age at baseline (years)** [a]			1.08 (1.07, 1.08)	1.08 (1.07, 1.08)	1.06 (1.06, 1.07)
**Age^2 at baseline** [b]			0.999 (0.999, 0.999)	0.999 (0.998, 0.999)	0.999 (0.998, 0.999)
**Sex (Ref: Female)**	Male		2.23 (2.12, 2.34)	2.22 (2.11, 2.33)	2.25 (2.14, 2.36)
**Ethnicity (Ref: White)**	South Asian			1.60 (1.34, 1.91)	1.40 (1.17, 1.67)
	Ethnic Minority Groups			1.05 (0.89, 1.24)	0.98 (0.82, 1.16)
**Country (Ref: England)**	Scotland			0.75 (0.70, 0.81)	0.78 (0.72, 0.83)
	Wales			0.77 (0.71, 0.84)	0.73 (0.68, 0.79)
**Townsend Deprivation Index (Ref: 1)**	2			1.10 (0.99, 1.23)	1.09 (0.98, 1.21)
	3			1.06 (0.95, 1.18)	1.03 (0.93, 1.15)
	4			1.15 (1.03, 1.28)	1.12 (1.00, 1.24)
	5			1.15 (1.03, 1.27)	1.09 (0.98, 1.21)
	6			1.13 (1.02, 1.26)	1.06 (0.95, 1.18)
	7			1.17 (1.05, 1.30)	1.07 (0.96, 1.19)
	8			1.28 (1.15, 1.42)	1.12 (1.01, 1.25)
	9			1.35 (1.21, 1.50)	1.12 (1.01, 1.25)
	10 (most deprived)			1.69 (1.52, 1.88)	1.24 (1.11, 1.38)
**No. of baseline conditions** [c]					1.18 (1.16, 1.20)
**No. of baseline conditions^2** [d]					0.995 (0.991, 0.998)
**Smoking (Ref: Never)**	Previous				1.09 (1.03, 1.15)
	Current				1.55 (1.44, 1.67)
**Alcohol intake (Ref: Daily or almost daily)**	Never				1.42 (1.29, 1.56)

Variables		Hazard Ratio (95% CI)			
		**Unadjusted**	**Model 1***	**Model 2****	**Model 3*****
	Special occasions only				1.33 (1.22, 1.45)
	1-3 times a month				1.23 (1.13, 1.35)
	1-2 times a week				1.18 (1.10, 1.27)
	3 or 4 times a week				1.03 (0.96, 1.10)
**Low physical activity (Ref: No)**	Yes				1.25 (1.17, 1.34)
**Sleep disturbance (Ref: Never/ Rarely)**	Sometimes				1.05 (0.99, 1.11)
	Usually				1.11 (1.04, 1.18)
**BMI (kg/m^2) (Ref: <25)**	25-29.9				1.31 (1.23, 1.39)
	30-34.9				1.51 (1.41, 1.62)
	≥35				1.61 (1.46, 1.76)

Abbreviation: BMI, Body Mass Index

[a] Scaled variable, age fitted as (age - mean age)

[b] Quadratic term for age

[c] Scaled variable, no. of baseline conditions fitted as (no. of baseline conditions – mean no. of baseline conditions)

[d] Quadratic term for the no. of baseline conditions

* Adjusted for age and sex

** Further adjusted for ethnicity, country, and Townsend Deprivation Index

*** Further adjusted for no. of baseline conditions, smoking, alcohol intake, low physical activity, insomnia, and BMI

Table S4: Hazard ratios for the association of baseline depression, age, sex, sociodemographic, baseline comorbidities, and lifestyle factors with incident PAD (n=167,125)

**Variables**		**Hazard Ratio (95% CI)**			
		**Unadjusted**	**Model 1***	**Model 2****	**Model 3*****
**Depression**		1.32 (1.15, 1.52)	1.58 (1.37, 1.83)	1.48 (1.28, 1.70)	0.89 (0.77, 1.04)
**Age at baseline (years)** [a]			1.11 (1.09, 1.12)	1.11 (1.10, 1.12)	1.09 (1.08, 1.10)
**Age^2 at baseline** [b]			0.999 (0.997, 1.000)	0.998 (0.997, 1.000)	0.998 (0.997, 1.000)
**Sex (Ref: Female)**	Male		2.38 (2.10, 2.70)	2.37 (2.09, 2.68)	2.07 (1.81, 2.35)
**Ethnicity (Ref: White)**	South Asian			1.56 (1.04, 2.35)	1.56 (1.03, 2.37)
	Ethnic Minority Groups			0.91 (0.59, 1.40)	0.87 (0.56, 1.35)
**Country (Ref: England)**	Scotland			0.90 (0.76, 1.07)	0.91 (0.77, 1.08)
	Wales			0.97 (0.80, 1.17)	0.88 (0.72, 1.06)
**Townsend Deprivation Index (Ref: 1)**	2			0.86 (0.63, 1.18)	0.84 (0.61, 1.15)
	3			1.05 (0.78, 1.43)	1.00 (0.74, 1.35)
	4			1.09 (0.80, 1.47)	1.02 (0.75, 1.38)
	5			1.28 (0.96, 1.71)	1.14 (0.85, 1.52)
	6			1.24 (0.93, 1.67)	1.05 (0.79, 1.42)
	7			1.51 (1.13, 2.01)	1.15 (0.86, 1.53)
	8			1.94 (1.48, 2.56)	1.36 (1.03, 1.79)
	9			2.19 (1.67, 2.88)	1.32 (1.00, 1.74)
	10 (most deprived)			3.31 (2.55, 4.30)	1.54 (1.18, 2.02)
**No. of baseline conditions** [c]					1.29 (1.24, 1.34)
**No. of baseline conditions^2** [d]					0.990 (0.984, 0.996)
**Smoking (Ref: Never)**	Previous				1.86 (1.59, 2.17)
	Current				6.88 (5.85, 8.08)
**Alcohol intake (Ref: Daily or almost daily)**	Never				1.32 (1.06, 1.63)

Variables		Hazard Ratio (95% CI)			
		**Unadjusted**	**Model 1***	**Model 2****	**Model 3*****
	Special occasions only				1.10 (0.89, 1.36)
	1-3 times a month				0.96 (0.76, 1.21)
	1-2 times a week				0.96 (0.81, 1.14)
	3 or 4 times a week				0.89 (0.74, 1.06)
**Low physical activity (Ref: No)**	Yes				1.52 (1.31, 1.76)
**Sleep disturbance (Ref: Never/ Rarely)**	Sometimes				1.00 (0.85, 1.17)
	Usually				1.07 (0.91, 1.26)
**BMI (kg/m^2) (Ref: <25)**	25-29.9				0.87 (0.75, 1.01)
	30-34.9				0.97 (0.82, 1.15)
	≥35				1.07 (0.86, 1.34)

Abbreviation: BMI, Body Mass Index

[a] Scaled variable, age fitted as (age - mean age)

[b] Quadratic term for age

[c] Scaled variable, no. of baseline conditions fitted as (no. of baseline conditions – mean no. of baseline conditions)

[d] Quadratic term for the no. of baseline conditions

* Adjusted for age and sex

** Further adjusted for ethnicity, country, and Townsend Deprivation Index

*** Further adjusted for no. of baseline conditions, smoking, alcohol intake, low physical activity, insomnia, and BMI

Table S5: Hazard ratios for the association of baseline depression, age, sex, sociodemographic, baseline comorbidities, and lifestyle factors with incident T2D (n=161,095)

Variables		Hazard Ratio (95% CI)			
		Unadjusted	Model 1*	Model 2**	Model 3***
**Depression**		1.27 (1.19, 1.35)	1.42 (1.34, 1.52)	1.37 (1.29, 1.46)	1.01 (0.94, 1.08)
**Age at baseline (years) [a]**			1.04 (1.03, 1.04)	1.05 (1.04, 1.05)	1.03 (1.03, 1.04)
**Age^2 at baseline [b]**			0.999 (0.999, 1.000)	0.999 (0.998, 0.999)	0.999 (0.999, 1.000)
**Sex (Ref: Female)**	Male		1.83 (1.74, 1.93)	1.82 (1.72, 1.91)	1.95 (1.84, 2.06)
**Ethnicity (Ref: White)**	South Asian			3.56 (3.11, 4.06)	3.23 (2.82, 3.71)
	Ethnic Minority Groups			2.49 (2.21, 2.82)	2.37 (2.09, 2.68)
**Country (Ref: England)**	Scotland			0.82 (0.76, 0.89)	0.83 (0.77, 0.90)
	Wales			1.12 (1.04, 1.21)	0.99 (0.91, 1.07)
**Townsend Deprivation Index (Ref: 1)**	2			0.95 (0.84, 1.09)	0.93 (0.82, 1.06)
	3			1.05 (0.93, 1.20)	0.98 (0.87, 1.12)
	4			1.15 (1.01, 1.31)	1.07 (0.94, 1.21)
	5			1.22 (1.08, 1.38)	1.07 (0.94, 1.21)
	6			1.30 (1.15, 1.47)	1.10 (0.98, 1.25)
	7			1.34 (1.19, 1.52)	1.08 (0.96, 1.23)
	8			1.56 (1.38, 1.75)	1.18 (1.05, 1.34)
	9			1.82 (1.61, 2.04)	1.25 (1.11, 1.41)
	10 (most deprived)			2.34 (2.08, 2.63)	1.41 (1.25, 1.59)
**No. of baseline conditions [c]**					1.13 (1.11, 1.15)
**No. of baseline conditions^2 [d]**					0.991 (0.988, 0.994)
**Smoking (Ref: Never)**	Previous				1.10 (1.04, 1.17)
	Current				1.53 (1.41, 1.65)
**Alcohol intake (Ref: Daily or almost daily)**	Never				1.67 (1.51, 1.85)

**Variables**		**Hazard Ratio (95% CI)**			
		**Unadjusted**	**Model 1***	**Model 2****	**Model 3*****
	Special occasions only				1.62 (1.48, 1.78)
	1-3 times a month				1.38 (1.25, 1.53)
	1-2 times a week				1.17 (1.07, 1.27)
	3 or 4 times a week				1.07 (0.98, 1.17)
**Low physical activity (Ref: No)**	Yes				1.25 (1.17, 1.34)
**Sleep disturbance (Ref: Never/ Rarely)**	Sometimes				1.12 (1.04, 1.20)
	Usually				1.17 (1.09, 1.26)
**BMI (kg/m^2) (Ref: <25)**	25-29.9				2.97 (2.68, 3.29)
	30-34.9				7.04 (6.35, 7.80)
	≥35				13.87 (12.44, 15.46)

Abbreviation: BMI, Body Mass Index

[a] Scaled variable, age fitted as (age - mean age)

[b] Quadratic term for age

[c] Scaled variable, no. of baseline conditions fitted as (no. of baseline conditions – mean no. of baseline conditions)

[d] Quadratic term for the no. of baseline conditions

* Adjusted for age and sex

** Further adjusted for ethnicity, country, and Townsend Deprivation Index

*** Further adjusted for no. of baseline conditions, smoking, alcohol intake, low physical activity, insomnia, and BMI

Table S6: Hazard ratios for the association of baseline depression, age, sex, sociodemographic, baseline comorbidities, and lifestyle factors with IBD (n=166,045)

**Variables**		**Hazard Ratio (95% CI)**			
		**Unadjusted**	**Model 1***	**Model 2****	**Model 3*****
**Depression**		1.30 (1.07, 1.58)	1.34 (1.10, 1.63)	1.30 (1.06, 1.58)	1.00 (0.81, 1.23)
**Age at baseline (years)** [a]			1.01 (1.00, 1.02)	1.02 (1.01, 1.03)	1.00 (0.99, 1.01)
**Age^2 at baseline** [b]			1.000 (0.999, 1.002)	1.000 (0.999, 1.002)	1.000 (0.999, 1.001)
**Sex (Ref: Female)**	Male		1.18 (1.00, 1.38)	1.17 (0.99, 1.37)	1.12 (0.95, 1.33)
**Ethnicity (Ref: White)**	South Asian			1.79 (1.08, 2.96)	1.79 (1.06, 3.00)
	Ethnic Minority Groups			1.00 (0.59, 1.68)	0.99 (0.59, 1.67)
**Country (Ref: England)**	Scotland			0.95 (0.75, 1.20)	1.00 (0.79, 1.26)
	Wales			1.08 (0.85, 1.38)	1.06 (0.83, 1.35)
**Townsend Deprivation Index (Ref: 1)**	2			1.41 (0.95, 2.11)	1.40 (0.94, 2.10)
	3			1.54 (1.04, 2.29)	1.52 (1.02, 2.25)
	4			1.37 (0.91, 2.07)	1.34 (0.89, 2.02)
	5			1.29 (0.86, 1.94)	1.25 (0.83, 1.88)
	6			1.52 (1.02, 2.26)	1.45 (0.97, 2.15)
	7			1.61 (1.08, 2.39)	1.48 (1.00, 2.21)
	8			1.74 (1.18, 2.58)	1.57 (1.06, 2.32)
	9			1.79 (1.21, 2.65)	1.53 (1.03, 2.27)
	10 (most deprived)			2.44 (1.67, 3.58)	1.92 (1.30, 2.84)
**No. of baseline conditions** [c]					1.16 (1.10, 1.22)
**No. of baseline conditions^2** [d]					0.998 (0.990, 1.007)
**Smoking (Ref: Never)**	Previous				1.53 (1.28, 1.82)
	Current				1.67 (1.30, 2.15)
**Alcohol intake (Ref: Daily or almost daily)**	Never				1.14 (0.82, 1.59)
	Special occasions only				1.34 (1.01, 1.78)

Variables		Hazard Ratio (95% CI)			
		**Unadjusted**	**Model 1***	**Model 2****	**Model 3*****
	1-3 times a month				0.82 (0.59, 1.14)
	1-2 times a week				1.04 (0.82, 1.32)
	3 or 4 times a week				1.01 (0.79, 1.30)
**Low physical activity (Ref: No)**	Yes				0.90 (0.70, 1.16)
**Sleep disturbance (Ref: Never/ Rarely)**	Sometimes				1.03 (0.84, 1.28)
	Usually				1.12 (0.89, 1.41)
**BMI (kg/m^2) (Ref: <25)**	25-29.9				0.97 (0.80, 1.18)
	30-34.9				0.98 (0.77, 1.24)
	≥35				0.87 (0.63, 1.21)

Abbreviation: BMI, Body Mass Index

[a] Scaled variable, age fitted as (age - mean age)

[b] Quadratic term for age

[c] Scaled variable, no. of baseline conditions fitted as (no. of baseline conditions – mean no. of baseline conditions)

[d] Quadratic term for the no. of baseline conditions

* Adjusted for age and sex

** Further adjusted for ethnicity, country, and Townsend Deprivation Index

*** Further adjusted for no. of baseline conditions, smoking, alcohol intake, low physical activity, insomnia, and BMI

Table S7: Hazard ratios for the association of baseline depression, age, sex, sociodemographic, baseline comorbidities, and lifestyle factors with incident PD (n=168,347)

Variables		Hazard Ratio (95% CI)			
		**Unadjusted**	**Model 1***	**Model 2****	**Model 3*****
**Depression**		1.29 (1.05, 1.57)	1.52 (1.25, 1.86)	1.53 (1.25, 1.87)	1.45 (1.18, 1.79)
**Age at baseline (years)** [a]			1.14 (1.12, 1.16)	1.14 (1.12, 1.16)	1.14 (1.12, 1.16)
**Age^2 at baseline** [b]			0.998 (0.996, 1.000)	0.998 (0.996, 1.000)	0.998 (0.996, 1.000)
**Sex (Ref: Female)**	Male		2.00 (1.69, 2.37)	2.00 (1.69, 2.37)	2.08 (1.74, 2.49)
**Ethnicity (Ref: White)**	South Asian			0.82 (0.34, 1.98)	0.60 (0.25, 1.47)
	Ethnic Minority Groups			1.54 (0.86, 2.73)	1.35 (0.76, 2.41)
**Country (Ref: England)**	Scotland			0.92 (0.72, 1.17)	0.93 (0.73, 1.18)
	Wales			0.94 (0.72, 1.21)	0.94 (0.72, 1.21)
**Townsend Deprivation Index (Ref: 1)**	2			0.74 (0.52, 1.04)	0.74 (0.52, 1.04)
	3			0.92 (0.66, 1.29)	0.92 (0.66, 1.28)
	4			0.95 (0.68, 1.33)	0.95 (0.68, 1.33)
	5			0.85 (0.61, 1.19)	0.85 (0.61, 1.19)
	6			0.61 (0.42, 0.89)	0.61 (0.42, 0.89)
	7			0.72 (0.50, 1.04)	0.73 (0.51, 1.05)
	8			0.83 (0.58, 1.19)	0.84 (0.59, 1.20)
	9			1.10 (0.79, 1.54)	1.12 (0.80, 1.57)
	10 (most deprived)			0.65 (0.44, 0.98)	0.67 (0.44, 1.00)
**No. of baseline conditions** [c]					1.03 (0.98, 1.09)
**No. of baseline conditions^2** [d]					1.005 (0.997, 1.013)
**Smoking (Ref: Never)**	Previous				0.82 (0.68, 0.97)
	Current				0.50 (0.34, 0.73)
**Alcohol intake (Ref: Daily or almost daily)**	Never				1.51 (1.10, 2.06)
	Special occasions only				1.16 (0.85, 1.58)

Variables		Hazard Ratio (95% CI)			
		**Unadjusted**	**Model 1***	**Model 2****	**Model 3*****
	1-3 times a month				1.23 (0.91, 1.67)
	1-2 times a week				0.95 (0.74, 1.22)
	3 or 4 times a week				0.97 (0.76, 1.25)
**Low physical activity (Ref: No)**	Yes				1.07 (0.81, 1.40)
**Sleep disturbance (Ref: Never/ Rarely)**	Sometimes				0.89 (0.73, 1.09)
	Usually				0.86 (0.68, 1.08)
**BMI (kg/m^2) (Ref: <25)**	25-29.9				1.21 (0.99, 1.48)
	30-34.9				0.99 (0.77, 1.28)
	≥35				0.82 (0.55, 1.22)

Abbreviation: BMI, Body Mass Index

[a] Scaled variable, age fitted as (age - mean age)

[b] Quadratic term for age

[c] Scaled variable, no. of baseline conditions fitted as (no. of baseline conditions – mean no. of baseline conditions)

[d] Quadratic term for the no. of baseline conditions

* Adjusted for age and sex

** Further adjusted for ethnicity, country, and Townsend Deprivation Index

*** Further adjusted for no. of baseline conditions, smoking, alcohol intake, low physical activity, insomnia, and BMI

Table S8: Hazard ratios for the association of baseline depression, age, sex, sociodemographic, baseline comorbidities, and lifestyle factors with incident IA (n=163,189)

Variables		Hazard Ratio (95% CI)			
		**Unadjusted**	**Model 1***	**Model 2****	**Model 3*****
**Depression**		1.40 (1.28, 1.53)	1.37 (1.25, 1.50)	1.37 (1.25, 1.50)	1.07 (0.97, 1.17)
**Age at baseline (years)** [a]			1.05 (1.05, 1.06)	1.06 (1.05, 1.06)	1.04 (1.03, 1.05)
**Age^2 at baseline** [b]			1.001 (1.000, 1.001)	1.001 (1.000, 1.001)	1.000 (1.000, 1.001)
**Sex (Ref: Female)**	Male		0.69 (0.64, 0.75)	0.69 (0.63, 0.74)	0.66 (0.61, 0.72)
**Ethnicity (Ref: White)**	South Asian			1.62 (1.22, 2.14)	1.46 (1.10, 1.95)
	Ethnic Minority Groups			1.24 (0.97, 1.60)	1.21 (0.94, 1.55)
**Country (Ref: England)**	Scotland			0.77 (0.68, 0.86)	0.80 (0.71, 0.90)
	Wales			0.88 (0.78, 0.99)	0.84 (0.75, 0.95)
**Townsend Deprivation Index (Ref: 1)**	2			0.89 (0.75, 1.04)	0.88 (0.75, 1.04)
	3			0.92 (0.78, 1.08)	0.90 (0.77, 1.06)
	4			0.98 (0.84, 1.16)	0.96 (0.82, 1.13)
	5			0.90 (0.76, 1.06)	0.87 (0.74, 1.02)
	6			0.87 (0.74, 1.03)	0.83 (0.70, 0.98)
	7			0.96 (0.81, 1.13)	0.88 (0.75, 1.04)
	8			0.96 (0.81, 1.14)	0.86 (0.73, 1.02)
	9			1.00 (0.84, 1.18)	0.85 (0.72, 1.00)
	10 (most deprived)			1.01 (0.85, 1.20)	0.79 (0.66, 0.94)
**No. of baseline conditions** [c]					1.18 (1.15, 1.21)
**No. of baseline conditions^2** [d]					0.992 (0.987, 0.996)
**Smoking (Ref: Never)**	Previous				1.12 (1.03, 1.21)
	Current				1.44 (1.27, 1.63)
**Alcohol intake (Ref: Daily or almost daily)**	Never				1.11 (0.95, 1.29)
	Special occasions only				1.09 (0.95, 1.25)

Variables		Hazard Ratio (95% CI)			
		Unadjusted	Model 1*	Model 2**	Model 3***
	1-3 times a month				0.95 (0.82, 1.10)
	1-2 times a week				0.95 (0.85, 1.07)
	3 or 4 times a week				0.99 (0.88, 1.12)
**Low physical activity (Ref: No)**	Yes				1.12 (1.00, 1.25)
**Sleep disturbance (Ref: Never/ Rarely)**	Sometimes				1.06 (0.96, 1.18)
	Usually				1.16 (1.04, 1.30)
**BMI (kg/m^2) (Ref: <25)**	25-29.9				1.14 (1.04, 1.26)
	30-34.9				1.20 (1.07, 1.34)
	≥35				1.33 (1.15, 1.54)

Abbreviation: BMI, Body Mass Index

[a] Scaled variable, age fitted as (age - mean age)

[b] Quadratic term for age

[c] Scaled variable, no. of baseline conditions fitted as (no. of baseline conditions – mean no. of baseline conditions)

[d] Quadratic term for the no. of baseline conditions

* Adjusted for age and sex

** Further adjusted for ethnicity, country, and Townsend Deprivation Index

*** Further adjusted for no. of baseline conditions, smoking, alcohol intake, low physical activity, insomnia, and BMI
